# Diagnostic Performance of Magnetic Resonance Sequences in Staging Lymph Node Involvement and Extranodal Extension in Head and Neck Squamous Cell Carcinoma

**DOI:** 10.3390/diagnostics15101251

**Published:** 2025-05-15

**Authors:** Giovanni Lorusso, Nicola Maggialetti, Francesca Laugello, Annalisa Garofalo, Ilaria Villanova, Sara Greco, Chiara Morelli, Pasquale Pignataro, Nicola Maria Lucarelli, Amato Antonio Stabile Ianora

**Affiliations:** Interdisciplinary Department of Medicine, Section of Radiology and Radiation Oncology, University of Bari “Aldo Moro”, 70124 Bari, Italy

**Keywords:** HNSCC, extranodal extension, lymph nodes, magnetic resonance imaging, magnetic resonance sequences

## Abstract

**Objectives:** This study aimed to evaluate the diagnostic performance of various MRI sequences in detecting nodal metastasis (N+) and extranodal extension (ENE+) in patients with head and neck squamous cell carcinoma (HNSCC). **Methods**: A retrospective analysis was conducted on 42 patients with HNSCC who underwent preoperative MRI and subsequent surgical lymph node dissection between June 2021 and December 2023. Lymph node MRI features were evaluated on five different MRI sequences. For each rN+ case, the presence of radiological extranodal extension (rENE+) was assessed independently in every MRI sequence by analyzing three characteristics. ENE was deemed positive if at least one of three criteria considered was present. **Results**: All of the MRI sequences demonstrated slightly high accuracy (~76%) for detecting N+, with T1WI, STIR, and CE THRIVE showing comparable sensitivities (60–65%). The STIR sequence exhibited the highest sensitivity (75%) and nearly the highest accuracy (91%) for detecting ENE+. Capsular irregularity and necrosis showed high specificity across sequences, while the loss of fatty hilum and nodal size had lower performance. **Conclusions**: Tailoring MRI protocols to leverage the strengths of specific sequences can significantly improve the diagnostic accuracy, aiding in better patient management and treatment outcomes.

## 1. Introduction

Head and neck squamous cell carcinoma (HNSCC) accounts for more than 90% of malignancies in the head and neck region and typically arises from the mucosal epithelium of the oral cavity, pharynx, or larynx [1,2].

Accurate lymph node assessment is essential for staging, treatment planning, and prognostication. Imaging plays a central role in detecting nodal metastases and extranodal extension (ENE), both of which significantly impact patient outcomes [3].

The 8th Edition of the American Joint Committee on Cancer (AJCC) staging system incorporated ENE into the N category [4], reflecting its strong prognostic value—except in HPV-related tumors, where its impact is less clear [5,6]. Reliable pre-treatment identification of ENE is therefore a clinical priority [7,8].

Various imaging modalities are used for lymph node assessment. Ultrasound is accessible and effective for superficial nodes, particularly when combined with fine-needle aspiration, but is operator-dependent and limited in deeper regions [9]. Computed tomography (CT) is widely used in clinical practice due to its high spatial resolution and rapid acquisition times, especially for evaluating the nodal size, morphology, and extracapsular spread [10], though its soft tissue contrast is inferior to magnetic resonance imaging (MRI). Positron emission tomography (PET)/CT provides functional data and is sensitive to small metastases, though it may produce false positives due to inflammation [11]. MRI, with its superior soft tissue contrast and advanced sequences, is particularly suited for evaluating deep cervical and retropharyngeal nodes, as well as perinodal infiltration [12].

The aim of this study was to analyze lymph nodes on magnetic resonance imaging (MRI) to identify which MRI sequences are the most sensitive in detecting nodal metastases (N+) and extranodal extension (ENE+), and to assess the diagnostic performance of individual MRI features.

## 2. Materials and Methods

### 2.1. The Study Population

Eighty-four consecutive patients with HNSCC undergoing surgical treatment with lymph node dissection at Policlinico of Bari between June 2021 and December 2023 were retrospectively enrolled to evaluate the sensitivity of each MRI sequence in establishing N+ and ENE+.

**Figure 1:**
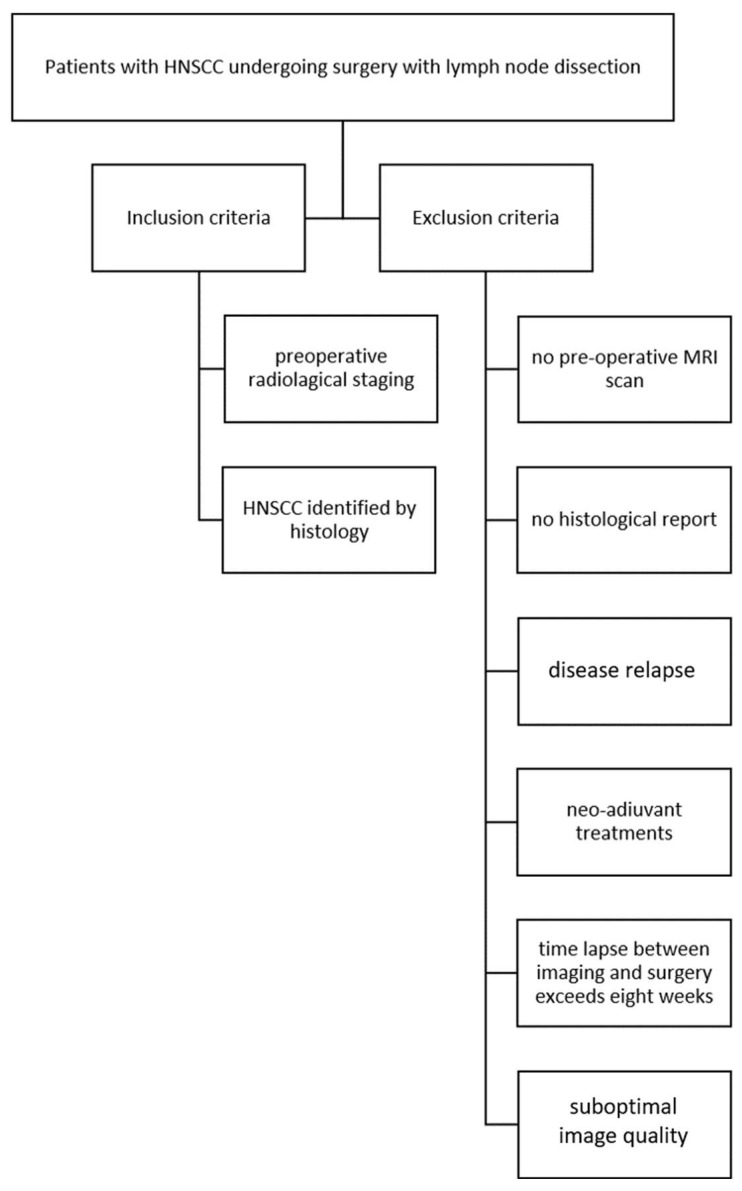
Inclusion and exclusion criteria flowchart.

Inclusion criteria were as follows: HNSCC identified with histology and preoperative radiological staging. Exclusion criteria were as follows: the absence of preoperative MRI, lack of histological report, patients with disease relapses, patients who underwent preoperative chemo-radiotherapy treatments, patients with more than eight weeks between MRI and surgery, and inadequate imaging quality (Figure 1).

For all patients, age, sex, and primary tumor site were collected from institutional electronic medical records.

### 2.2. MRI Scans

The MRI examinations were performed using a 1.5 Tesla scanner (Philips Achieva Nova Dual, Philips Medical Systems, Best, The Netherlands) equipped with a phased array surface coil. The images were acquired from the base of the skull to the apex of the lung. The MRI protocol used was coronal and axial T1-weighted turbo spin echo (TSE), axial and sagittal T2-weighted TSE, axial short tau inversion recovery (STIR), axial volumetric, fat–saturated, gradient echo T1-weighted sequence (T1 High-Resolution Isotropic Volume Excitation, THRIVE), and axial contrast-enhanced THRIVE (Table 1). DWI was acquired with b values of 0 s/mm and 1000 s/mm. Extracellular gadolinium-based contrast media (Gadovist, Bayer Pharma AG, Berlin, Germany) was administered at a weight-adjusted dose of 0.1 mmol/kg at a flow rate of 2 mL/s.

All of the MRI images were also archived using our institutional PACS (Enterprise Imaging, Biesse Medica, Roma, Italy).

### 2.3. Imaging Assessment

Two head and neck radiologists (N.M., 12 years of experience; A.A.S.I., 25 years of experience) analyzed, retrospectively, the MRI images independently, and were blinded to patient pathological results about nodal and extranodal involvement; disagreements were resolved by open discussion. Whenever feasible, three target lymph nodes were chosen. For each of these lymph nodes, the MRI images were evaluated for the presence or absence of round shapes, the loss of fatty hilum, necrosis, and irregular capsules, and the short-axis diameter was measured. These MR imaging characteristics were evaluated independently on T1-weighted, T2-weighted, STIR, THRIVE, and CE THRIVE. 

**Figure 2:**
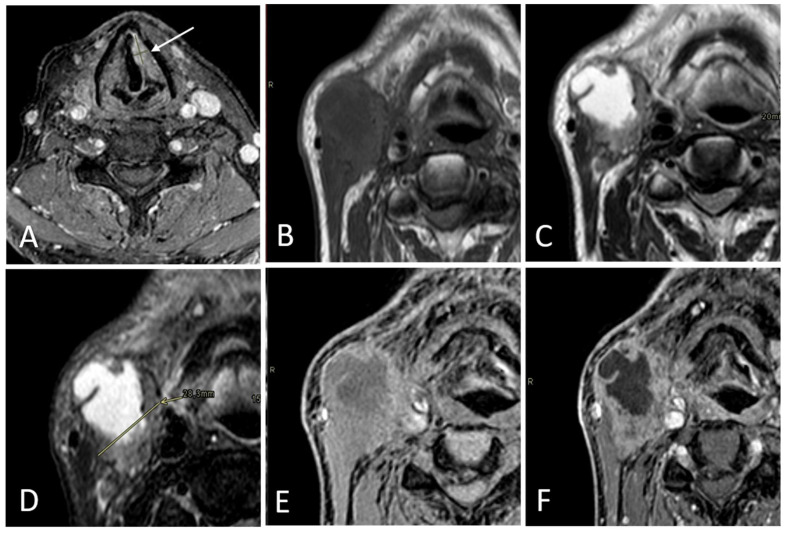
Right-sided level IB lymph node in a 70-year-old male patient with a laryngeal tumor, evaluated as part of a pre-treatment assessment. The primary tumor (arrow) is shown in (**A**). The SAD measures 28 mm. Loss of fatty hilum, necrosis, and a round shape are well detectable in T1WI (**B**), T2WI (**C**), STIR (**D**), THRIVE (**E**), and CE THRIVE (**F**). Capsular irregularity with fat invasion and gross muscle/vessel invasion is shown in all of the sequences. Pathologists reported this lymph node as N+ and ENE+.

**Figure 3:**
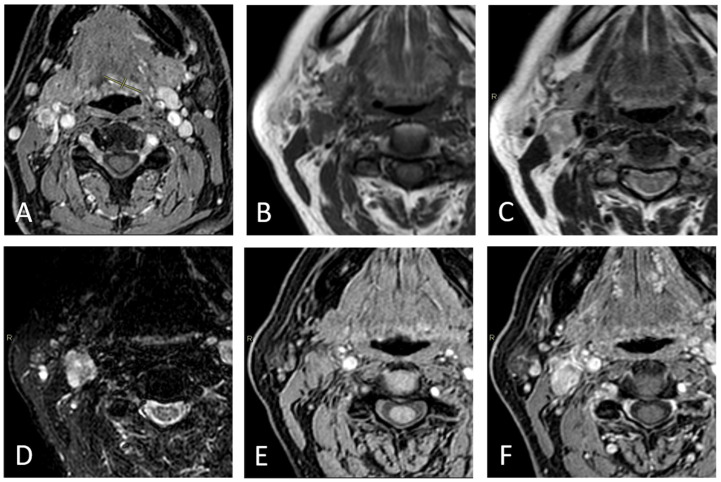
Right-sided level IIA lymph node in a 63-year-old male patient with a tongue tumor, evaluated as part of a pre-treatment assessment. The primary tumor is shown in (**A**). The SAD measures 12 mm. No necrosis, fatty hilum, or round shape are detectable in T1WI (**B**), T2WI (**C**), STIR (**D**), THRIVE (**E**), and CE THRIVE (**F**). No capsular irregularity is shown in any sequence. Pathologists reported this lymph node as N-.

**Figure 4:**
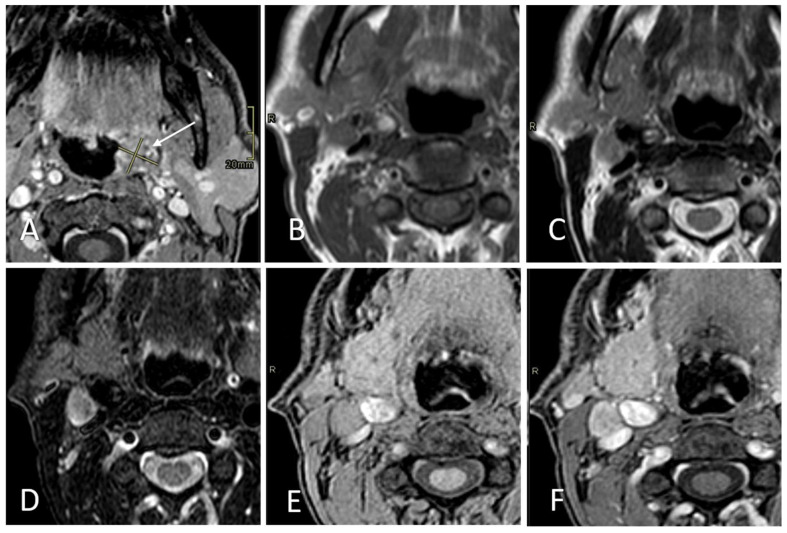
Right-sided level IIA lymph node in a 34-year-old female patient with a tongue tumor, evaluated as part of a pre-treatment assessment. The primary tumor is shown in (**A**). The SAD measures 13 mm. Necrosis is shown in T2WI (**C**) and STIR (**D**). Loss of fatty hilum and round shape are detectable in T1WI (**B**), T2WI (**C**), STIR (**D**), THRIVE (**E**), and CE THRIVE (**F**). No capsular irregularity is shown in any sequence. Pathologists reported this lymph node as N+ and ENE-.

According to Node-RADS 1.0 [13], the lymph node diameter was considered normal when its short-axis diameter (SAD) measured less than 10 mm. Exceptionally, a cutoff of less than 5 mm in the SAD was applied for facial, parotid, retroauricular, occipital, retropharyngeal, and anterior jugular lymph nodes. Nodes characterized by the presence of at least two out of five features were considered radiologically metastatic (rN+). For each rN+ case, the presence of rENE+ was assessed independently in every MRI sequence by analyzing the following three characteristics: capsular irregularity with fat stranding, capsule irregularity with fat invasion, and gross muscle/vessel invasion; ENE was deemed positive if at least one of these criteria was present according to the literature [14] (Figure 2, Figure 3 and Figure 4).

### 2.4. Statistics

Statistical analysis was performed using SPSS software (version 26.0 SPSS Inc., Armonk, NY, USA). Descriptive statistics, including means, standard deviations (SDs), and percentages, were used to summarize the data. Inter-reader agreement between the two radiologists was assessed using the intraclass correlation coefficient (ICC). The sensitivity, specificity, positive and negative predictive value, and accuracy of the selected MRI criteria were evaluated in each sequence for the detection of N+ and ENE+, using histopathology as the gold-standard reference.

The diagnostic accuracy of each MRI sequence in detecting N+ and ENE+ was assessed by a receiver operating characteristic (ROC) analysis.

## 3. Results

A total of 84 patients with HNSCC undergoing surgical treatment with lymph node dissection were evaluated; 36 patients were excluded for the unavailability of MRI preoperative imaging; 5 patients were excluded for the unavailability of the histological report; 1 patient was excluded due to an interval between imaging and surgery higher than eight weeks.

The mean acquisition time was 23 min and 30 s, and no adverse reactions were observed in any patients following the contrast injection.

A total of 42 patients were enrolled in this study. A total of 18/42 (42.86%) patients were female and 24/42 (57.14%) were male; the mean age was 66 (SD 14) and the average interval between the MRI and surgery was 4,6 weeks (SD 2.6).

No patients were excluded due to poor image quality.

Primary tumor sites included the oral cavity in 6 patients (14%), nasopharynx in 9 patients (21%), hypopharynx in 25 patients (60%), and larynx in 2 patients (45%) (Table 2).

A total of 118 lymph nodes were evaluated, of which 26/118 (22.03%) were pathological N+ and 8/118 (7.78%) were pathological ENE+.

Overall inter-reader agreement between the two head and neck radiologists for the assessment of nodal imaging features, as well as for the evaluation of rENE+ across all MRI sequences, was excellent, with an ICC of 0.897.

The sensitivity, specificity, PPV, NPV, and accuracy of rN+ and rENE+ for each sequence are reported in Table 3. Diagnostic accuracy tests for predicting N+ and ENE+ were also evaluated for each radiologic feature, considering every MRI sequence independently (Table 4 and Table 5).

## 4. Discussion

Several studies have highlighted the prognostic value of N+ and ENE+ [15,16,17], leading to the inclusion of ENE in the regional lymph node staging for HNSCC. Currently, Node-RADS is recommended for lymph node assessment; contrariwise, the literature lacks consensus on imaging criteria for assessing ENE+. The gold standard for N+ and ENE+ diagnosis is the histopathological evaluation of excised lymph nodes. Nonetheless, employing imaging for the pre-treatment detection of N+ and ENE+ could allow us to plan the best treatment options before the initial intervention. As indicated by recent meta-analyses, CT and MRI demonstrate a comparable diagnostic performance in detecting ENE in HNSCC, without significant differences in the pooled sensitivity and specificity [18,19,20]. The primary goal of this study was to assess the diagnostic performance of the MRI sequences used in the HNSCC protocol (T1WI, T2WI, STIR, THRIVE, and CE THRIVE) in detecting N+ and ENE+. To evaluate N, we carefully selected five features based on a thorough literature review [14,21,22], i.e., the loss of fatty hilum, necrosis, a round shape, capsular irregularity, and the short-axis diameter. We excluded the feature of heterogeneous enhancement from this study, since it is not part of the Node-RADS criteria [13] and is only evaluable using the CE THRIVE sequence, and so is not compatible among the other sequences. In our study, for N+ detection, all of the sequences showed a slightly high accuracy, and the T1WI, STIR, and CE THRIVE sequences showed comparable sensitivities. In particular, the low variability in sensitivity indicates the complexity of identifying metastatic lymph nodes, which often requires a combination of imaging features. A round shape, capsular irregularity, and necrosis showed high rates of specificity and accuracy in detecting N+ in all sequences, except necrosis, which is poorly assessed in the T1 sequence, because necrotic tissue does not have sufficiently distinct T1 relaxation properties compared to the surrounding tissues, leading to a low signal difference. The T2-weighted and CE THRIVE sequences were better at highlighting pathological changes like necrosis; the T2-weighted images showed areas of increased fluid content, which is typical in necrosis, as the hyperintense and contrast-enhanced images showed necrotic tissue as ipointense, but not enhanced. In addition, our results showed the low performance of the loss of fatty hilum across all MRI sequences, likely due to MRI’s limited ability to assess this feature in small lymph nodes. The current understanding suggests that the lymph node size is not a reliable predictor of metastases; enlarged nodes are often hyperplastic rather than metastatic, and nodes smaller than 10 mm can still harbor micrometastases [17]. Furthermore, the Node-RADS indications specify that facial, parotid, retroauricular, occipital, retropharyngeal, and anterior jugular lymph nodes are considered enlarged if their size is 5 mm or greater. Subsequently, this study has assessed the diagnostic ability of each MRI sequence to detect the criteria of rENE+. Significant disagreement exists in the literature regarding which imaging criteria should be used for evaluating ENE+ [23,24]. Based on the most commonly used criteria, our study considered capsular irregularity with fat stranding, with fat invasion, or with muscle/vessel invasion. The STIR sequence showed the highest sensitivity and almost the highest accuracy for ENE detection. This aligns with previous studies suggesting the superiority of STIR sequences in highlighting the soft tissue signal and detecting pathological changes [25]. CE THRIVE resulted in the second most performing sequence; contrast medium could help in distinguishing between lymph nodes and extranodal adipose tissue. All of the MRI features, in every sequence studied to predict ENE+, showed very high values of specificity and NPV, but low values of sensitivity; these features could be highly reliable for confirming the absence of a pathological condition, but not for detecting its presence, for which histopathology examination remains mandatory. Both in detecting N+ and ENE+, every sequence singly had low rates of PPV but high rates of NPV. All of the sequences were individually effective at ruling out a pathological condition compared to confirming it, implying that it should be used with caution in scenarios with positive results that need further verification, i.e., histopathological examination. The ROC analysis further confirmed the diagnostic accuracy of each MRI sequence. The AUC for detecting N+ was the highest for the T1WI and CE THRIVE sequences, followed closely by STIR. For ENE+, the STIR sequence had the highest AUC, indicating its superior diagnostic performance compared to the other sequences. Future research could focus on integrating advanced imaging techniques, such as dynamic contrast-enhanced MRI, diffusion-weighted imaging (DWI), and the apparent diffusion coefficient (ADC), to further enhance the diagnostic accuracy. Furthermore, larger prospective studies are needed to validate these findings and explore their impact on clinical outcomes. In addition to the selection of MRI sequences, the integration of artificial intelligence (AI) and radiomics into radiological diagnostics presents a promising advancement [25]. These algorithms can be trained on large datasets to recognize patterns associated with N+ and ENE+, thereby improving the diagnostic consistency and reducing inter-observer variability. According to our results, the development of a structured diagnostic flowchart integrating the most sensitive and specific MRI sequences could help to guide radiologists in the evaluation of nodal and extranodal involvement. Our study has some limitations. Firstly, the study was conducted retrospectively, which might introduce selection bias. The patients were selected based on the availability of preoperative MRI imaging and histological reports, potentially excluding a subset of patients who might have influenced the results differently. Future studies with larger cohorts are necessary to validate our results and provide more robust data. It is possible that the lymph node assessed by the radiologist was not the same one evaluated by the pathologist, given that multiple lymph nodes can be present at the same level. Our imaging was conducted within 8 weeks before the neck dissection; thus, ENE could potentially develop during the interval between imaging and dissection. The spatial resolution of MRI could represent a technical limitation, particularly for assessing small lymph nodes [18,26].

## 5. Conclusions

MRI plays a critical role in the preoperative evaluation of HNSCC, particularly for the assessment of ENE. This study confirms that specific sequences offer distinct advantages in detecting N+ and ENE+. In our study, all of the sequences demonstrated a slightly high accuracy, and the T1WI, STIR, and CE THRIVE sequences showed comparable sensitivities for N+ detection, while the STIR sequence exhibited the highest sensitivity and nearly the highest accuracy for ENE detection. Tailoring MRI protocols and developing a structured diagnostic flowchart that leverages the strengths of each sequence can significantly improve the diagnostic accuracy and patient management in HNSCC.

## Figures and Tables

**Figure 5:**
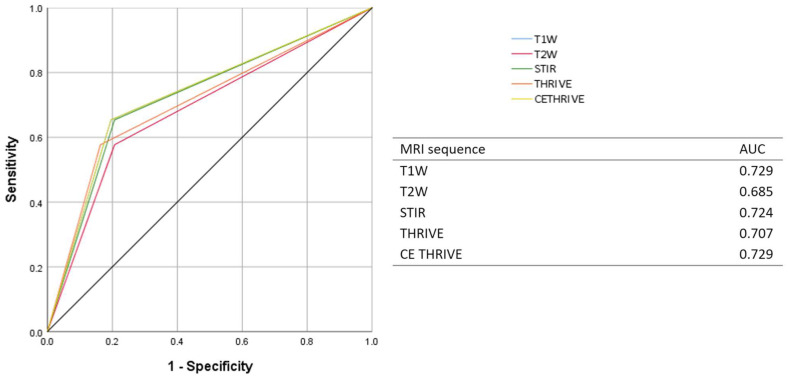
ROC curve and AUC of each MRI sequence for detecting N+.

**Figure 6:**
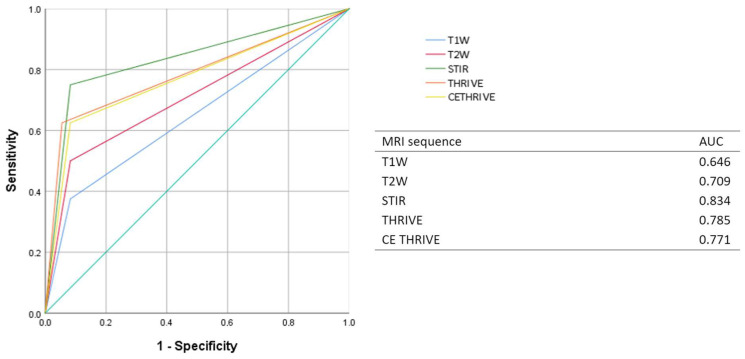
ROC curve and AUC of each MRI sequence for detecting ENE+.

**Table 1:** Details of the study protocol pulse sequence parameters.

Parameter	T1WI Axial	T1WI Coronal	T2WI Axial	T2WI Sagittal	STIR Axial	THRIVE	CE THRIVE
Sequence type	TSE	TSE	TSE	TSE	IR	GRE 3D	GRE 3D
TR/TE (ms)	650/20	650/20	4000/80	4000/80	2100/70	8/4	8/4
Fat suppression	No	No	No	No	Yes	Yes	Yes
Thickness (mm)	4	4	4	4	4	1.5	1.5
Acquisition time	3′25″	3′25″	3′30″	3′30″	3′40″	3′00″	3′00″

**Table 2:** Patient characteristics and tumor site.

Patient Characteristics	N = 42 Patients
Sex	
Male; Female	24 (57%); 18 (42%)
Age (mean; SD)	66; 14
Primary tumor site	
Oral cavity	6 (14%)
Nasopharynx	9 (21%)
Hypopharynx	25 (60%)
Oropharynx	0 (0%)
Larynx	2 (5%)

**Table 3:** The sensitivity, specificity, PPV, NPV, and accuracy of single sequences in the evaluation of radiological N+ and ENE+.

		Sensitivity	Specificity	PPV	NPV	Accuracy
T1WI	N+	65%	80%	49%	89%	77%
	ENE+	38%	92%	25%	95%	88%
T2WI	N+	58%	79%	44%	87%	75%
	ENE+	50%	92%	31%	96%	89%
STIR	N+	65%	79%	47%	89%	76%
	ENE+	75%	92%	40%	98%	91%
THRIVE	N+	58%	84%	50%	88%	78%
	ENE+	63%	95%	45%	97%	92%
CE THRIVE	N+	62%	84%	48%	88%	78%
	ENE+	63%	94%	42%	97%	92%

**Table 4:** The sensitivity, specificity, PPV, NPV, and accuracy of MRI characteristics of N+ for each sequence.

		Sensitivity	Specificity	PPV	NPV	Accuracy
T1WI	Loss of fatty hilum	62%	59%	30%	84%	59%
	Necrosis	8%	97%	40%	79%	77%
	Round shape	50%	93%	68%	87%	84%
	Capsular irregularity	42%	95%	69%	85%	83%
	Short-axis diameter	54%	61%	28%	82%	59%
T2WI	Loss of fatty hilum	54%	57%	26%	81%	56%
	Necrosis	15%	98%	67%	80%	80%
	Round shape	38%	93%	63%	84%	81%
	Capsular irregularity	35%	95%	64%	84%	81%
	Short-axis diameter	54%	61%	28%	82%	59%
STIR	Loss of fatty hilum	58%	64%	31%	84%	63%
	Necrosis	27%	95%	58%	82%	80%
	Round shape	42%	93%	65%	85%	82%
	Capsular irregularity	42%	95%	69%	85%	83%
	Short-axis diameter	54%	61%	28%	82%	59%
THRIVE	Loss of fatty hilum	46%	67%	29%	82%	63%
	Necrosis	12%	97%	50%	79%	78%
	Round shape	38%	93%	63%	84%	81%
	Capsular irregularity	35%	95%	64%	84%	81%
	Short-axis diameter	54%	61%	28%	82%	59%
CE THRIVE	Loss of fatty hilum	50%	58%	25%	80%	56%
	Necrosis	31%	95%	62%	83%	81%
	Round shape	46%	93%	67%	86%	83%
	Capsular irregularity	38%	93%	63%	84%	81%
	Short-axis diameter	54%	61%	28%	82%	59%

**Table 5:** The sensitivity, specificity, PPV, NPV, and accuracy of MRI characteristics of ENE+ for each sequence.

	Imaging Characteristics of ENE+					
	Capsular Irregularity	Sensitivity	Specificity	PPV	NPV	Accuracy
T1WI	Fat stranding	13%	100%	100%	94%	94%
	Fat invasion	0%	95%	0%	93%	89%
	Muscle/vessel invasion	25%	95%	29%	95%	91%
T2WI	Fat stranding	13%	100%	100%	94%	94%
	Fat invasion	13%	94%	13%	94%	88%
	Muscle/vessel invasion	25%	97%	40%	95%	92%
STIR	Fat stranding	13%	100%	100%	94%	94%
	Fat invasion	38%	95%	33%	95%	91%
	Muscle/vessel invasion	25%	97%	40%	95%	92%
THRIVE	Fat stranding	13%	99%	50%	94%	93%
	Fat invasion	38%	96%	43%	95%	92%
	Muscle/vessel invasion	25%	98%	50%	95%	93%
CE THRIVE	Fat stranding	25%	98%	50%	95%	93%
	Fat invasion	13%	94%	13%	94%	88%
	Muscle/vessel invasion	25%	98%	50%	95%	93%

ROC analysis was performed to assess the diagnostic accuracy of each MRI sequence in detecting N+ and ENE+. The AUCs of T1WI, T2WI, STIR, THRIVE, and CE THRIVE were, respectively, 0.694, 0.656, 0.705, 0.686, and 0.710 in assessing N+, and 0.646, 0.709, 0.834, 0.785, and 0.771 in assessing ENE+ (Figure 5 and Figure 6).

## Data Availability

The original contributions presented in the study are included in the article, further inquiries can be directed to the corresponding author.

## Abbreviations

HNSCC	head and neck squamous cell carcinoma
STIR	short tau inversion recovery
THRIVE	T1WI High-Resolution Volume Excitation
TSE	turbo spin echo
IR	inversion recovery
GRE	gradient echo
TR	repetition time
PPV	positive predictive value
NPV	negative predictive value
CE	contrast-enhanced
WI	weighted imaging
ENE+	extranodal extension
N+	nodal involvement
SAD	short-axis diameter
ROC	receiver operating characteristic
AUC	area under the curve
